# Addressing the Research and Development Gaps in Modern Phage Therapy

**DOI:** 10.1089/phage.2023.0045

**Published:** 2024-03-18

**Authors:** Paul E. Turner, Joana Azeredo, Ed T. Buurman, Sabrina Green, Jakob Krause Haaber, Douglas Haggstrom, Koichi Kameda de Figueiredo Carvalho, Claas Kirchhelle, Mercedes Gonzalez Moreno, Jean-Paul Pirnay, Mirza Alas Portillo

**Affiliations:** ^1^ Department of Ecology and Evolutionary Biology, Yale University, New Haven, Connecticut, USA.; ^2^ Microbiology Program, Yale University School of Medicine, New Haven, Connecticut, USA.; ^3^ Center for Phage Biology and Therapy, Yale University, New Haven, Connecticut, USA.; ^4^ Centre of Biological Engineering, University of Minho, Braga, Portugal.; ^5^ LABBELS—Associate Laboratory, Braga, Guimarães, Portugal.; ^6^ CARB-X, Boston University, Boston, Massachusetts, USA.; ^7^ Laboratory of Gene Technology, Department of Biosystems, KU Leuven, Leuven, Belgium.; ^8^ SNIPR Biome ApS, Copenhagen, Denmark.; ^9^ INCubator for Antibiotic Therapies Europe (INCATE), Basel, Switzerland.; ^10^ Innovation Office, University of Basel, Basel, Switzerland.; ^11^ Centre Emile Durkheim, University of Bordeaux, Talence, France.; ^12^ School of History, University College Dublin, Dublin, Ireland.; ^13^ Leibniz Institute for Natural Product Research and Infection Biology, Hans Knoll Institute, Jena, Germany.; ^14^ Laboratory for Molecular and Cellular Technology, Queen Astrid Military Hospital, Brussels, Belgium.

**Keywords:** antimicrobial resistance, biofilm, biotechnology, patient access, personalized medicine

## Abstract

Antimicrobial resistance is on the rise globally, prompting increased research and development (R&D) of phage therapy as a strategy to address difficult-to-treat bacterial infections. We review the current state of phage therapy research, including major operational, epistemic, and biological challenges for phage R&D, and discuss some new approaches to developing the technology motivated by recent breakthroughs such as artificial intelligence and synthetic phage production. In addition, we contextualize these R&D challenges and opportunities in light of the ongoing predicament of commercial antimicrobial innovation and current public–private efforts to reinvigorate the pipeline of antimicrobial drug discovery. We conclude with reflections on the potential for new phage therapies to be readily accessible across all income contexts to better ensure broad patient access, and consider possible alternatives to current public and public–private solutions for phage therapy and production.

## Introduction

Phages are ubiquitous in the biosphere and are constantly changing through evolution to improve their infectivity on bacteria, particularly lytic phages that are selected to efficiently “prey” upon host cells. For this reason, phages are attractive as potential antimicrobials, and in principle evolution should continuously supply novel candidate pools of phages with therapeutic usefulness.^1^ The history of the co-discovery of phages by Frederick Twort and Félix d'Hérelle in 1915 and 1917 and the early development of phage-based applications, especially phage therapy and prevention, have been reviewed extensively.^2–5^

This rich history includes early uses of phages in the 1920s to “type” (specifically identify) which bacterial strains were responsible for epidemic disease spread, and phage production in the 1920–1940s in countries such as Belgium, France, Great Britain, Germany, and the United States as therapies targeting bacterial diseases in humans and domesticated animals.^6^ It is seldom acknowledged that Belgian scientists played a prominent role in developing phage culture techniques widely used today, such as double-agar overlays to visualize plaques and René Appelmans' protocol to expand phage host-range, as well as early clinical trials and published phage therapy studies.^7–11^

Notably, many of these early efforts preceded the licensing of the sulfonamide prontosil in 1935, and the subsequent widespread use of chemical antibiotics in Western medicine, ca. World War II and afterward.^12^ Although improved postwar access to antimicrobials of broader spectrum led to a pronounced loss of interest in phage therapy, phages remained a popular therapeutic option in areas where antimicrobials were scarce.

In the USSR and other Eastern bloc countries, research on phage applications continued—although improved antimicrobial access in these locales also led to a reduction of usage.^13^ Today, the alarming rise in antimicrobial-resistant (AMR) infections and a dwindling pipeline of newly identified antibiotic candidates place phages firmly in the spotlight, creating resurged interest in the promise of phage therapy to address the global public health crisis of AMR infections.^14–16^

In this study, we survey the current state of phage therapy research, including major operational, epistemic, and biological challenges for phage research and development (R&D) alongside new opportunities emerging from current breakthroughs in artificial intelligence (AI) and synthetic phage production. We next contextualize these R&D challenges and opportunities in light of the ongoing crisis of commercial antimicrobial innovation and current public–private efforts to reinvigorate the antimicrobial pipeline. We end by reflecting on how potential new phage therapies can be made accessible across all income contexts, and consider potential alternatives to current public and public–private solutions for phage therapy and production.

## Key R&D Challenges

Myriad research challenges exist whenever new medical therapies are under development (or older approaches are recycled; see Research article 2 in SI), especially when seeking generalized approaches that transcend borders and address different regulatory hurdles. In the case of phage therapy, the host-specificity of lytic phages poses unique challenges when it comes to developing effective interventions that do not select for resistance, enable researchers to gather robust safety and efficacy data, and are scalable for use outside of specialized settings.

As highlighted by recent reviews of phage therapy R&D, the resulting biological, epistemic, and operational hurdles are amplified by popular perceptions of phages as “biological drugs” that should be used and evaluated according to the regulatory, clinical, and market frameworks developed for chemotherapy.^17^

### *À la carte* versus *prêt-à-porter*

In general, phage ability to infect is limited to a subset of genotypes of a single target bacterial species, which is different from a narrow- or especially broad-spectrum antibiotic that can negatively impact growth of multiple pathogens (and unfortunately many commensals in the microbiome). To deal with the phage specificity hurdle, two distinct—yet compatible—phage therapy approaches have been developed.^18^

In what could be called the *prêt-à-porter* approach, defined broad-spectrum phage cocktails are applied, which are supposed to target most bacteria suspected to cause certain infectious diseases. In *à la carte* phage therapy concepts, one or more phages are chosen from a phage bank, or taken from the environment, to efficiently target the patient's infecting bacteria.^19^

Although both approaches are patient-centric, the *à la carte* approach has clinical advantages when it comes to choosing an optimized treatment regime for a sick individual.^20–22^ It also promises to be more sustainable when it comes to preventing bacterial resistance. As with antibiotics, selection pressure exerted by sustained mass-application of lytic phages—as may result from a *prêt-a-porter* phage therapy model—may lead to bacterial resistance against therapeutic phage(s), with observed outcomes in emergency phage therapy.^23,24^

Although numerous proposals have been advanced to minimize the chance of treatment failure such as to steer bacteria to evolve phage resistance that compromises pathogenicity traits (i.e., evolutionary trade-offs), the *à la carte* approach for targeting only the specific infecting bacterial genotype could diminish selection pressure for widespread evolution of phage resistance.^25–27^ This would reduce the probability for a broad mechanism of resistance to evolve and spread through horizontal gene transfer, and decrease the risk it would persist in the environment and in clinical settings.^28,29^

However, the *à la carte's* approach to biological specificity comes at an epistemic and operational cost. Epistemically, treatments vary in indications, target pathogens, treatment regimens (dose and duration), and concomitant antibiotic therapy, making it difficult to draw general conclusions on efficacy and develop scalable protocols. Personalized approaches are also more time-consuming and logistically complex than one-size-fits-all approaches, necessitating more frequent production of numerous different phage stocks and the exchange of bacterial strains and personalized phage preparations between phage manufacturers and health care recipients.

In addition, such personalized or precision medicine concepts are, in general, not compatible with conventional medicinal product development and licensing pathways which require several years and considerable investments to complete.^30^

### (Dys)synergistic interactions

In addition to the strategic R&D challenge of deciding between *à la carte* or *prêt-à-porter* approaches, researchers also face considerable uncertainty about how their phages will work when administered alongside conventional chemical antimicrobials and in the context of complex microbial biofilms.

Although popular histories tend to focus on the golden era of heroic phage therapy experimentation between the 1920s and 1940s (see Research article 2 in SI), the historical record shows that phages were rarely used as stand-alone therapeutics. Ongoing evaluation of 1010 historical French phage therapy records from 1945 to 1990 indicates that the majority of clinical uses of phages occurred alongside rather than instead of antibiotics.^31^

More recent studies show that combined treatment using phage(s) together with antibiotic(s) can lead to synergy that improves treatment outcomes.^32^ Thus, it is plausible that phage-use in therapy could be alongside (i.e., as adjuvant with) currently approved antibiotics, and developed/marketed accordingly. The phrase “Make Antibiotics Great Again” (S. Green, personal communication) comes from this concept of bringing back antibiotics that have been considered “useless” due to the ongoing antibiotic-resistance crisis by combining them with phage and making them effective again.

However, we note that phage-antibiotic synergy is a phenomenon where *in vitro* studies indicate particular phage–bacteria–antibiotic genotype × genotype × environment interactions can matter for efficacy.^33^ These uncertainties suggest that testing for positive versus negative phage/antibiotic synergy should be required before each personalized treatment.^34^

Biofilm-associated infections have traditionally posed another biological challenge for phage therapy. For instance, some phages are less capable of effectively targeting bacteria when they grow in biofilms or on mucosal surfaces.^35–37^ However, the treatment time buffer of these usually chronic infections has also allowed for significant progress in the development and preparation of personalized therapeutic strategies.^38^

Recent successfully treated clinical cases involved the use of inhaled phage therapy targeting pathogenic *Pseudomonas aeruginosa* and *Achromobacter* bacteria.^39,40^ A 2022 review of phage therapy against biofilm infections in clinical case reports spanning 2018–2021 described a positive outcome in 96% of cases with complete resolution of the bacterial infection in 67% of patients.^41^ Similar outcomes have been reported in other studies.^42^

Although these results are promising, data should be interpreted carefully because of the heterogeneity among studies, which used differing routes of administration and phage concentrations; variable applications (single vs. multiple phages); and most importantly, combination therapy with antibiotics or other treatments in most cases, creating uncertainty over the success attributable to phage(s). Moreover, the majority of these studies do not provide a thorough evaluation of patient healing, particularly analysis of the bacterial and phage populations during treatment. Such data would be particularly useful for revealing the true impact of phages in treating clinical biofilms.

Despite limited confirmatory data from clinical cases, *in vitro* studies performed in controlled laboratory conditions have helped elucidate phage/biofilm interactions, providing some useful guidance in translational development of phage treatment in clinical practice. Some phages have intrinsic properties that confer advantages for controlling biofilms.^38^

A recent systematic analysis of *in vitro* phage/biofilm interaction indicates that higher phage concentrations are strongly associated with improved biofilm degradation, and phages with larger burst sizes and shorter latent periods seem to be particularly good candidates for killing biofilms.^43^ Nevertheless, the protective effect of the biofilm matrix and the rapid proliferation of phage-resistant bacterial mutants pose serious challenges for using phages as stand-alone therapeutics.

This protective effect suggests that phages combined with other chemical and/or mechanical treatments may be necessary for best efficacy against chronic biofilms. For example, it is observed that the biofilm matrix of *Staphylococcus epidermidis* protects bacteria against lytic phage predation, such that mechanical dispersion of the biofilm produces considerably enhanced phage efficacy.^44^ Case reports of phage therapy against recalcitrant implant-associated infections have documented potential beneficial outcomes when phages are applied after debridement of the prosthesis that harbors the biofilm.^45^

As stated earlier, phages can also synergize with chemical antibiotics; this interaction may be useful in biofilm control. A potential mechanism for this is that because some phages can rapidly reduce biofilms causing cell destruction and dispersion, this may release nutrients and reactivate dormant bacteria, thus enhancing antibiotic efficacy against physiologically active cells. If phage-resistant mutants proliferate, these genotypes may be more susceptible to antibiotics due to evolved trade-offs.^46^

Some studies have identified the importance of differing sequential treatment when both phages and antibiotics are deployed; for example, initial application of phages followed by subsequent administration of antibiotics led to improved biofilm eradication.^47,48^ When the treatment order was reversed, however, biofilm exposure to antibiotics preceding phage deployment resulted in antagonistic interactions for some combined treatments.^48^ These observations highlight the importance of performing *in vitro* studies, which predict the best phage/antibiotic combinations that should minimize the risk of such antagonistic effects.

Another strategy to improve phage efficacy is to evolve phages in the laboratory, so they are preadapted to better degrade bacteria present in biofilms (or indeed for other phenotypes).^19^ Such preadapted (or “trained”) phages can delay the development of evolved phage-resistant bacteria. One example where this was carried out attributed the improvement in the efficacy of phages to the fact that they had evolved the ability to utilize two cellular receptors, whereas untrained (wild-type) phages were confined to binding through only a single receptor.^49^

Because biofilm populations are typically heterogeneous, they can encompass bacterial strains that vary in their susceptibilities to attack by therapeutic phages. Through the *in vitro* adaptation process, evolutionary training of phages can select for viruses with a broader host range and, therefore, greater ability to infect a wider diversity of host cells in the biofilm population, reducing the proliferation of phage-resistant variants that permit biofilm regrowth.

Greater efforts are needed to measure phage efficacy against biofilms, particularly when combining phages with other treatments, to systematically assess relevant scenarios in the clinic. Although valuable knowledge is gained from phage–biofilm interactions under controlled laboratory conditions, these likely fail to mimic the complexities of *in vivo* environments. Clearly, development of phage therapy would also benefit from increased attention to defining standardized and reproducible methods for rigorously assessing phages as anti-biofilm agents, to best harness this technology when targeting chronic AMR biofilm infections.

### A role for newer technology such as AI?

The arrival of increasingly powerful technologies such as AI and synthetic biology could help mitigate many of the outlined phage specificity and evolved phage resistance concerns.^50–52^ In addition to fundamentally restructuring the role of intellectual property (IP) regimes shaping current phage banks (see Research article 4 in SI), both technologies could be integrated to create single point-of-care devices, enabling the timely identification and production of personalized phage preparations.^53^

Imagine a device where a patient infecting sample is loaded, the DNA is extracted and sequenced, and an AI-driven algorithm connects to a database that predicts the phage (natural or synthetic) genome most likely to lyse the infecting bacteria. This is followed by synthesis of the phage genome(s), and production (transcription and translation) using a cell-free particle assembly process.^54^ Phages are then mixed with bacterial isolate in a validation module, to confirm *in vitro* efficacy.

Although hypothetical, it is worth highlighting that such a device would have considerable advantages over classically produced phages; foremost, it eliminates the need to maintain therapeutic phage-bank libraries (see Research article 4 in SI), and avoids dispatching the patient's bacterial sample and the therapeutic phage match to a possibly far-off location. In addition, on-site synthesized phage production would be possible against bacteria that eminently jeopardize public health or which constitute bioterrorist threats.^55^ Currently, phage therapy targeting potentially lethal bacteria must be grown under high biosafety level (e.g., BSL-3) and strict biocontainment conditions.

However, synthetic production could leverage phages synthesized at BSL-1. Also, by avoiding phage propagation on bacteria in the laboratory, the genome sequences of viruses identified in metagenomics data could be produced, to benefit wider exploration of possible phage candidates in the biosphere.^56^ Last, phage preparations made through cell-free synthesis contain fewer (or zero) potentially harmful molecules that can negatively impact a patient, particularly endotoxins; and the device conceivably would produce phages even when humans are entirely isolated for long or short periods, such as during space travel or while living in submarines.^57^

This imagined device would represent a paradigm shift for phage therapy ambitions, moving from the medicinal-product market to the health care-device market. Whereas today's industry is striving to develop and market defined phage products, this futuristic “phage printer” example would require interdisciplinary expertise of both scientists and engineers, representing diverse fields such as design methods, laboratory automation, micro-/nanofluidics, magnetic levitation, microelectronics, nanotechnology, AI, synthetic biology, information and communications technology (ICT), and diagnostics.

The products will no longer be the phages; instead, these would be the phage bio-ink reagents (enzymes, nucleotides, amino acids, and ATP) used to produce synthetic phages; the AI algorithm; and the access to regularly updated phage/bacteria interaction databases.

## Translating Research into Therapy

Although AI and synthetic phage production may lead to major paradigm shifts, major economic challenges for phage therapy remain. One of the most pronounced is to develop economically sustainable and socially accessible ways of making effective phage therapy available across the three One Health sectors of human, animal, and environmental health. As outlined hereunder, phages' host-specificity makes them sit uneasily within traditional anti-infective business models. Marketing opportunities have been further impacted by the ongoing decline of commercial antimicrobial innovation. Clearly before this might become a reality, the AI algorithms need encompass data from ecological studies on medically relevant pathogens, as these data are needed to identify and predict which features are related to efficacy.

Since the 1980s, there has been a major and consistent slowdown of private sector investment in antimicrobial innovation. A 2020 survey of the pharmaceutical industry showed that only six large multinational pharmaceutical companies remain active in antibiotic research.^58^ With traditional Big Pharma actors abandoning the field, antimicrobial R&D has been concentrated in relatively smaller- and medium-sized companies that face substantial commercial pressures to assure high profitability margins for their investors while competing with more lucrative therapeutic areas.^59^

Meanwhile, scientists in government laboratories and at academic institutions have also continued to pursue discovery and development of novel antibiotics, with a subset leading an expanding decentralized network of specialized phage therapy centers.

### Public phage development: magisterial preparations and personalized treatments

The decline of actors capable of bench-to-bedside development and large-scale commercial rollouts has forced phage researchers to consider alternative development models. One such model involves the creation of dedicated public or academic phage therapy centers providing personalized treatments for local and regional patients. In response to growing concerns about AMR and phage therapy's potential, academic institutions are creating centers for phage research that may help address a critical void left unfilled by industry.

Belgium's phage therapy program was established in 2008, after phage treatments in 2007 at the military hospital in Brussels.^60,61^ More recently, TAILOR at Baylor College of Medicine (Houston, TX) was developed as an end-to-end service center providing phages to hospitals in the largest medical center in the world and to other major clinical partners, such as the Mayo Clinic.^62^ Other centers in the United States strive to cover all or part of the R&D pipeline spanning phage discovery to patient therapy, including IPATH Center for Innovative Phage Applications and Therapeutics (UC San Diego School of Medicine) and the Yale Center for Phage Biology & Therapy (Yale University).

Centers dedicated to phage therapy R&D have also been established in countries, including Australia, Canada, France, Germany, the United Kingdom, and Israel, whereas long-standing ones still operate in locations such as Poland and the Republic of Georgia. As such dedicated centers become more numerous, there is increasing likelihood that personalized phage treatments should be accessible to patients in nearby hospitals in their vicinity.

A major advantage of this decentralized emergence of public phage therapy lies in centers' ability to harness multiple sources of funding, network effectively when it comes to sharing expertise and phages, and reduce reliance on traditional industry investment streams. Although important issues surrounding the bioethics and accessibility of publicly developed therapies for lower income patients require more attention (Lewis et al., in preparation), centers can receive support from academic institutions, external research funders, private philanthropy, and hospitals using the services provided by these centers.

Similar to the historical business model of Institut Pasteur, another source of revenue for ongoing public innovation could lie in the licensing of innovations or patentable products that could be developed while manufacturing phage treatments or in creating monopoly supply contracts for certain regions.^63^

Belgium's “Phage Valley” illustrates one model for this concept of public phage development and supply for a national health care system. In the Belgian regulatory framework, phages can be delivered in the form of magistral preparations (equivalent to compounding pharmacy preparations in the United States) to the patient upon prescription by the treating physician and preparation by the hospital pharmacy.^64^

The phages are the active pharmaceutical ingredients, which are prepared according to a monograph by a dedicated center (Queen Astrid Military Hospital—QAMH, Brussels, Belgium) and that undergo independent quality checks by the Scientific Institute of Public Health (Sciensano).^65^ Through this agreement, personalized phage therapy treatments become possible for patients. Notably, Belgium has been innovating the field of phage therapy through intensive international collaboration with other phage centers.

This involvement of phage experts in the founding of centers is not new, evidenced by the model of the Eliava Institute (Tbilisi, Georgia), where phage biologists George Eliava and Félix d'Hérelle were key individuals who founded this longest-operating phage therapy institute in the world. To date, roughly 150 personalized phage therapy cases have been facilitated by the QAMH group with high rates of success, largely due to leveraging the aforementioned phage/antibiotic synergies when treating patients. QAMH also has supplied phages to multiple centers throughout the world and shared expertise in many aspects of phage selection, production and delivery.

### For-profit phage development: biotechs, funders, and coordinators

In addition to the rapid growth of academic and nonprofit phage R&D, recent years have also seen a significant increase of interest in phage therapy by biotechnology companies.^66^ However, the need for costly clinical trials, and the fact that phage therapy is not fully approved in any high-income country (HIC) pose major hurdles for commercial developers.^67^ Sharply rising international concerns about AMR have seen numerous public and nonprofit interventions emerge to address “market failures” and align public health needs to commercial interests.^68,69^

According to the Global AMR Hub's Dynamic Dashboard (June 11, 2023), governments and nongovernmental funders have invested in >506 different phage-related R&D projects since 2010 with at least 14 resulting phage preparations undergoing clinical trials.^70^ In the following, we use the examples of two nonprofit initiatives as well as that of a Danish biotech currently developing a novel phage cocktail to discuss attempts to establish a “commercial phage pipeline” and ongoing challenges of for-profit phage R&D.

One of the most important challenges for commercial phage developers is securing sustained funding both for their high-risk preclinical R&D and the substantial investments involved in scaling up production for clinical trials. Launched in 2021, INCATE (Incubator for Antibacterial Therapies in Europe) advances early-stage ventures through strategic, R&D, regulatory and other advice, preparing companies for larger nondilutive and equity financing.^71^ Roughly 10% of the innovators contacting INCATE since its launch (∼220 total) are focused on phage therapeutics and INCATE has started working with 11 number of phage developers.

Access to financially sustainable and nondilutive funding is of crucial importance to the current ecosystem of mostly smaller companies. Over recent years, CARB-X (Combating Antibiotic-Resistant Bacteria Accelerator) has emerged as one of the main nonprofit funders to derisk preclinical innovation and help companies trial products. CARB-X was initiated in 2016 in the United States to provide financial, scientific, business, and regulatory support to groups developing novel antimicrobial therapeutics, diagnostics, and preventatives.

CARB-X is funded by three governments: the Biomedical Advanced Research and Development Authority (BARDA) in the United States, the United Kingdom (UK) Department of Health and Social Care's Global Antimicrobial Resistance Innovation Fund (GAMRIF), and Germany's Federal Ministry of Education and Research (BMBF). Support also comes from the Wellcome Trust and the Bill & Melinda Gates Foundation. In addition, CARB-X receives in-kind support from the National Institute of Allergy and Infectious Diseases (NIAID), part of the US National Institutes of Health (NIH).

In 2019, a specific CARB-X call was aimed at nontraditional modalities, which included phage therapy. Funding supported four phage projects aimed at treating or preventing infections. By advancing phage molecular biology tools, these projects aim to foster rational design and engineering of phages to improve bacterial killing while circumventing some drawbacks of naturally occurring therapeutic phages (e.g., narrow host range, development of phage resistance, and poor efficacy in biofilm killing).^51^

Phico Therapeutics explores use of their small acid-soluble spore protein (SASP) technology in therapy against *P. aeruginosa*; here, the phage is used to precisely deliver a gene to the pathogen genome to express a protein preventing bacterial replication. Locus Biosciences uses a CRISPR-engineered Phage (crPhage), LBP-KP01, against *Klebsiella pneumoniae* to complement a similar cocktail against *Escherichia coli* previously evaluated in a Phase-IB trial in patients with a history of urinary tract infections.

Eligo Bioscience utilizes CRISPR technology to uniquely remove extended-spectrum-β-lactamase and carbapenemase genes within commensal strains of *K. pneumoniae* and *E. coli* in the gastrointestinal (GI) tract of transplant patients. The fourth CARB-X supported project was SNIPR001, a phage cocktail developed by Danish company SNIPR Biome that targets *E. coli* in the GI tract, as an orally administered prophylactic to protect against *E. coli* blood-stream infections.^72^

SNIPR Biome created its cocktail by first isolating and screening candidate wild-type phages. This was followed by design-build-test-learn iterations to engineer useful phage traits, including complementing tail-fibers, CRISPR-Cas kill circuits, and optimized expression of those elements from biofilm active promoters.

A lead panel of engineered CRISPR-armed phages then underwent chemistry, manufacturing, and control (CMC), comprehensive *in vitro* testing, and *in vivo* pharmacokinetics/pharmacodynamics to yield the assembled SNIPR001 cocktail of four complementing CRISPR-armed phages.^72^ Scale-up depended on developing consistent production of a pharmaceutical-grade SNIPR001 cocktail. This included creation of “master” stocks of phages and bacteria cell banks to reduce variability when propagating purified phages.

For a Phase-1 clinical trial, the SNIPR001 material was manufactured in 15 L fermenters, which should be further scalable for subsequent trials. Standard CMC protocols dictate preservation of product-component stability over time; SNIPR Biome measured the concentrations of each CRISPR-armed phage at both drug-substance and drug-product stages to detect any stability changes during 24 months of storage. To assure continuity of engineered phage components, test standards were devised based on whole-genome sequencing of specimens.

In view of U.S. Food and Drug Administration requirements that phage therapeutics are analyzed for genes conferring DNA integration, AMR, virulence, and transduction, *in silico* analysis of SNIPR001 phage genomes confirmed that the CRIPSR-armed phages lacked any known transposase or integrase genes. Findings implied that the SNIPR phages are not temperate and, therefore, unlikely to be capable of integrating into the chromosomes of host bacteria.

In addition, there was no evidence that phage genomes contained genes homologous to those associated with AMR or virulence. Last, the analyses confirmed that SNIPR001 CRISPR-armed phages did not cause generalized transduction of bacteria when tested *in vitro*.^72^

Numerous commercial phage products have undergone clinical trials, whereas still more are underway (e.g., NCT052277350, NCT03808103, NCT04684641, NCT05010577, and NCT05488340).^73–76^ Overall, between 2000 and 2015, clinicaltrials.gov recorded merely 7 phage therapy trials; whereas, in 2022 alone, 18 new trials were initiated, showcasing a notable surge in both private and health systems interest in phage-based products.^77^

As of March 2023, the listed trials in clinicaltrials.gov reached 45, with 5 in the advanced phase-III stage.^77^ However, in the absence of major pharmaceutical reinvestment in antimicrobial development, coordination services and nonprofit funding from organizations such as INCATE and CARB-X will likely continue to play a vital role in sustaining the emergent ecosystem of small for-profit phage developers.

## Access

The outlined biological, economic, and regulatory challenges facing phage R&D are exacerbated by questions about how to make resulting products accessible across all income contexts. Although AMR poses a substantial threat to health and food production systems globally, lack of access to any effective antibacterials remains one of the primary health challenges in low-income contexts. Entities such as CARB-X strive to ensure broad access to their funded products, but greater attention of industry could be devoted to addressing this challenge.^78^

Although existing public and academic centers play an important role in the regional provision of personalized phage therapy, they remain primarily located in high- or medium-income metropolitan hubs. Efforts are underway to form larger phage networks such as the African Phage Forum which is mainly at the research and discovery phase. Reflecting on how translational phage research could move forward involves considering different aspects and models of production, particularly in the perspective of public health.

As the case of SARS-CoV-2 vaccines has shown, equity and public interest need to be considered as fundamental drivers of the many steps of pharmaceutical development if we want to effectively address global health care issues.^79,80^

A first point to consider is the IP management. Despite uncertainty over patentability of phages isolated from nature, phage therapy development might result in IP rights for genetically modified phages, or for phage therapy-related innovations such as diagnostics.^81^ To avoid that patents and other exclusive rights affect future R&D and pose problems to accessibility of treatments and diagnostics, phage development initiatives may consider different IP management strategies.

This could involve the adoption of a nonpatenting policy of innovations, as did the FACT Consortium that developed fixed dose combinations against malaria, ASAQ and ASMQ, or does the Mario Negri Institute of Pharmacological Research.^82^ Alternatively, it could adopt a socially responsible IP management in product development partnerships.

Pursuing the protection of research outputs might be discussed case by case, for instance, as a strategy to assure accessibility of the end-products, where one example is the Drugs for Neglected Diseases Initiative (DNDi)'s IP approach and that of the closely related Global Antibiotic Research and Development Partnership (GARDP). It could also involve implementing a policy of sharing knowledge assets (data, technologies, or viruses), or through initiatives such as patent pools.^83^

A second concern is phage production. As innovations for which private industry is still reluctant to invest, phage therapeutics could be conceived through alternative models of production, particularly through support from public funding. The article has already outlined some initiatives of phage production by European and US public hospitals.^17^ However, outside of these HIC contexts, phage production could take into consideration other experiences of public health-driven manufacturing.

One example is Brazil, where the State supports a set of pharmaceutical laboratories that produce vaccines, generic drugs, and diagnostic kits to supply the government's pharmaceutical policies.^84^ Similar to recent models proposed for antibiotic R&D, phage therapy production could be implemented through know-how transfer and national or regional infrastructures that take into account non-HIC health care needs and access to end-products in the technological development.^63,85^

In general, the re-emergence of interest in phage therapy could present an opportunity to learn from drug-development models that emerged from scenarios where there was little commercial interest. For example, tuberculosis, malaria, and neglected diseases are health care challenges where public, private, and philanthropic financing have been leveraged to develop therapies that can be provided at affordable prices.^59^ Phage therapy is unlikely to replace antibiotics completely, particularly if research on phage-antibiotic synergy suggests these combinations prove useful in the clinic.

However, given the need to approve as many therapeutic options as possible for addressing the growing threat of AMR, this offers an opportunity to pursue approaches outside of the traditional pathways and to circumvent the commercialization pressures which antibiotics face in the market today.

## Concluding Remarks: Building a Sustainable Phage Value Chain

Recent studies on the social return on investment in phage therapy highlight a social economic need and benefit for the technology.^86^ But, if regulation is not aligned with the distinctive features of phage therapy, the commercial approval and use of phages likely will face the same profitability, microbial resistance, and access issues as those hindering novel antibiotics.

In view of the global scale of the AMR challenge, it is likely that both non- and for-profit models as well as *à la carte* and *prêt-à-porter* models will play important roles going forward. Phages would also sit well within “delinked” volume-independent reimbursement models for novel antimicrobials or indirect commercial benefits such as patent-exclusivity vouchers. These “pull” models have been discussed in depth during the past decade, leading to initial implementations in countries such as the United Kingdom, whereas other nations have initiated discussion on their legislation (e.g., United States and Australia) or agreements among member states (e.g., European Union).

However, these are early steps and many relevant questions remain unanswered as the phage therapy industry development continues to evolve. Are there ample resources (whether public or private-equity funding) in the immediate future to conduct a sufficient number of well-controlled clinical studies that establish safety and show efficacy that proves the unique benefits of phage therapy? Can a sustainable market be achieved for novel innovative alternatives to chemical antibiotics—or different models that can drive this innovation forward? Is there sufficient good manufacturing practice capacity available at reasonable costs to allow innovative phage therapy strategies to be examined in clinical trials?

Should the value chain for phage therapeutics be centralized or modular, and could a hybrid model involving public–private partnerships prove viable? How does the therapeutic use relate to other potential markets for phage products, such as infection prevention, targeted therapeutic delivery (e.g., microbiome modulation), and applied uses of phage subproducts such as endolysins? How will potential breakthrough technologies be made accessible to users across high-, medium-, and low-income contexts? And how to foster the latter's participation in manufacturing, for instance, through technology transfer?

The future of phage therapy lies in continuous exploration, innovation, and collaboration among stakeholders as well as the creation of durable infrastructures facilitating knowledge production and exchange. Both current decentralized public options and for-profit startups applying both *à la carte* and *prêt-à-porter* are well-positioned to drive progress in the field due to their experimental nature and enthusiasm. Only by addressing critical questions such as those posed earlier, and through lessons learned from a variety of current and historical profit and nonprofit models, can we expect the phage therapy sector to achieve sustainable growth and significantly impact AMR infections, ultimately benefiting public health.
